# Organization of Paris Hospitals

**Published:** 1844-01-13

**Authors:** 


### ORGANIZATION OF PARIS HOSPITALS.

The organization of the Paris hospitals is justly considered, by all those who are acquainted with it, to be such as to render them far more efficient in relieving the wants of the poor, and in contributing to the advancement of science, than our own charitable institutions of a similar nature. But, howsoever we may admire the real charity displayed in their management, and the liberal spirit of their medical regulations; yet, on a close inspection, we find imperfections in the governing body which often nullify, to a certain extent, the very regulations to which we allude. The sources from whence the Paris hospitals derive their income are twofold; the rental of the private property belonging to the hospitals, amounting to £250,000, and a subsidy, or kind of poor-rate, furnished by the city of Paris, also amounting to £250,000. The entire management and control of this income of half a million sterling, as also the nearly irresponsible government of the hospitals, is vested in a Board, or Council, composed, entirely, with one exception, of legal and civic functionaries: The Dean of the Faculty of Medicine is the one in whose favour an exception to this systematic exclusion of the medical profession has been made. Thus, in a body of sixteen individuals intrusted with the government of all the medical charities of a city, the population of which numbers at least a million of souls, the medical interests of the inhabitants of those charities, and the personal interests of those whose knowledge is called upon to alleviate their sufferings, are entrusted to a single physician, who, however willing, however energetic, generally finds himself powerless and alone amongst so many. The result of such an organization of the governing body may easily be imagined. The members of the Board, looking upon the medical functionaries of the hospitals as persons employed under them, adopt every possible means of making the physicians and surgeons as dependant as possible on the administration, every now and then reminding them, rather harshly, of their relative positions, if they become at all "meddlesome." Thus, in order to ensure more completely this dependance, and to prevent anything like violent opposition on the part of its medical subordinates, as it considers them, the Board has taken care to insert, as a clause in the nomination of the surgeons and physicians, although that nomination takes place by concours, that it will be renewed by the Board every five years, if the Board be satisfied with the previous conduct of the nominee. This clause is rarely, if ever, resorted to in order to sanction the dismissal of a medical officer; but the Board takes care to renew the nomination at the stated periods with sufficient solemnity to strike terror into the breasts of those who have any inclination to be "factious." The physicians and surgeons, therefore, are generally as submissive and silent a body as the Board could wish, each being individually afraid to advance, lest he should endanger his own safety.—Their equanimity has, however, been lately sorely put to the trial by a gross invasion of their rights, and their anger has at last got the better of their discretion.

The concours has now been adopted for many years, as the sole means of deciding the appointments of physicians and surgeons to the Paris hospitals.—The successful candidates at the concours constitute a kind of supplementary body, under the name of "Bureau Central," from which, in regular rotation, the vacant appointments are filled. As, however, when an appointment in one of the central hospitals becomes vacant, those who are already placed in the hospitals of the suburbs are entitled to take it, the young physicians and surgeons are nearly all at first located in the most eccentric hospitals, and only gradually arrive at the more desirable appointments, after a series of severe ordeals, and years passed in attending hospitals that are situated at a distance from town, at great personal inconvenience. Instigated, probably, by private influence more than by a desire to relieve suffering humanity, or to advance science, the governing Board has lately given to M. Guerin, the well-known orthopædist, several wards at the Hôpitaux des Enfans, to enable him, they say, to carry on his experiments in that branch of science. Unfortunately, M. Guerin does not belong to the hospitals, not being a surgeon of the "Bureau Central," nor is in any other way connected with the administration of the hospital. The Board has also given to M. Le Roy d'Etiolles, also well known to the profession by his writings on lithotrity, permission to give gratuitous advice to those among the poor who are affected with urinary diseases who apply for relief at the general receiving rooms of the administration. *Inde viæ;* the young surgeons who form the "Bureau Central," considering these appointments an invasion of their rights, and also an infraction of the concours system, first sounded the alarm, several energetic appeals, both to the medical and non-medical public, being made by them on the subject, through the medium of the press. The seniors of the profession have not. however, left them alone to defend the system to which they nearly all owe their professional eminence, and last week the "Gazette des Hôpitaux" contained the protestations of MM. Marjolin, Velpeau, Lisfranc, Berard, Laugier, and Ricord against the recent appointments.

It is impossible to say what will be the result of these energetic protestations, addressed, as they are, to a non-medical body, but we may nearly calculate on their inefficacy as regards any modification in the appointments which have already been made. It is, however, to be hoped that, seeing what a strenuous opposition the medical body makes to this infringement of their rights, the Board will hesitate again to incur the odium which attaches itself to such censures.

A remarkable feature in this contest is the great hostility shown by the Paris surgeons to "specialities." The admission which the "specialists" have obtained into the medical bodies by these appointments is universally deplored. This feeling is not peculiar to our brethren of Paris; for illustrious men of all ages have continually looked upon "specialism" in disease as nearly inseparable from quackery, and, generally in proportion as they have been supported by the public, they have been anathematised by the profession. In this instance we see that it is a non-medical Board which forcibly imposes "specialities" on the medical public; at the same time it is regretted that such really superior men as M. Guerin and M. Le Roy d'Etiolles should have placed themselves in the position which they at present occupy, by applying for, or accepting, appointments to which they are considered to have no claim.—*Loudon Lancet*, Oct. 21, 1843.

